# Molecular Evidence for Two Domestication Events in the Pea Crop

**DOI:** 10.3390/genes9110535

**Published:** 2018-11-06

**Authors:** Oldřich Trněný, Jan Brus, Iveta Hradilová, Abhishek Rathore, Roma R. Das, Pavel Kopecký, Clarice J. Coyne, Patrick Reeves, Christopher Richards, Petr Smýkal

**Affiliations:** 1 Agricultural Research Ltd., 66441 Troubsko, Czech Republic; trneny.oldrich@gmail.com; 2 Department of Geoinformatics, Palacký University, 783 71 Olomouc, Czech Republic; jan.brus@upol.cz; 3 Department of Botany, Palacký University, 783 71 Olomouc, Czech Republic; hradilovai@seznam.cz; 4 The International Crops Research Institute for the Semi-Arid Tropics, Hyderabad, Telangana 502324, India; a.rathore@cgiar.org (A.R.); r.das@cgiar.org (R.R.D.); 5 Crop Research Institute, The Centre of the Region Haná for biotechnological and Agricultural Research, 783 71 Olomouc, Czech Republic; kopecky@genobanka.cz; 6 United States Department of Agriculture, Washington State University, Pullman, WA 99164-6402, USA; Clarice.coyne@ars.usda.gov; 7 United States Department of Agriculture, National Laboratory for Genetic Resources Preservation, Fort Collins, CO 80521, USA; pat.reeves@ars.usda.gov (P.R.); chris.richards@ars.usda.gov (C.R.)

**Keywords:** domestication, Ethiopian pea, pea, *Pisum sativum*, seed dormancy

## Abstract

Pea, one of the founder crops from the Near East, has two wild species: *Pisum sativum* subsp. *elatius*, with a wide distribution centered in the Mediterranean, and *P. fulvum,* which is restricted to Syria, Lebanon, Israel, Palestine and Jordan. Using genome wide analysis of 11,343 polymorphic single nucleotide polymorphisms (SNPs) on a set of wild *P. elatius* (134) and *P. fulvum* (20) and 74 domesticated accessions (64 *P. sativum* landraces and 10 *P. abyssinicum*), we demonstrated that domesticated *P. sativum* and the Ethiopian pea (*P. abyssinicum*) were derived from different *P. elatius* genepools. Therefore, pea has at least two domestication events. The analysis does not support a hybrid origin of *P. abyssinicum*, which was likely introduced into Ethiopia and Yemen followed by eco-geographic adaptation. Both *P. sativum* and *P. abyssinicum* share traits that are typical of domestication, such as non-dormant seeds. Non-dormant seeds were also found in several wild *P. elatius* accessions which could be the result of crop to wild introgression or natural variation that may have been present during pea domestication. A sub-group of *P. elatius* overlaps with *P. sativum* landraces. This may be a consequence of bidirectional gene-flow or may suggest that this group of *P. elatius* is the closest extant wild relative of *P. sativum*.

## 1. Introduction

About 10,000 years ago, humans began the domestication of crops and animals, initiating one of the largest changes in human history. There are two broad views of the origin of plant cultivation and domestication, especially in the Near East. One proposes that crops’ origin was singular, rather fast (hundreds of years) and took place in the so-called small core area of south-eastern Turkey and adjoining parts of Syria [1], followed by further evolution of domesticated crops that improved their quality [2]. The alternative view is that domestication was a protracted process with multiple origins of crops that went on slowly and in parallel in multiple locations [3,4,5,6,7,8]. Recent studies propose that the use of wild cereals and pulses considerably predate their domestication [3,4,9]; similarly, the gathering of pea from ‘gardens’ by pre-agricultural peoples has been discussed [10].

The Near Eastern center of domestication has been extensively studied [11,12] and plant domestication in this region has provided a large number of crops that are important today: cereals: einkorn wheat (*Triticum monococcum* L.), emmer wheat (*Triticum dicoccum* (Schrank) Schuebl), barley (*Hordeum vulgare* L.), fibre and oil crop: flax (*Linum usitatissimum* L.) and legumes: lentil (*Lens culinaris* Medic), chickpea (*Cicer arietinum* L.), bitter vetch (*Vicia ervilia* L.) Willd., faba bean (*Vicia faba* L.) and pea (*Pisum sativum* L.) [1,9,12,13,14,15,16,17,18]. In early agriculture and until the widespread use of N-fertilizer from the Haber-Bosch process [19,20], grain legumes accompanied cereals [2,9,13]. Indeed, the co-domestication of cereals and legumes was a recurring feature in the independent origins of agriculture [21]. Despite the crucial position of legumes as an important source of protein in the human diet, comparatively little is known about their domestication.

The legume family is one of the most abundantly represented among crops. Although cereals have a higher total production, there are at least as many legume crops. FAOstat lists 12 cereal crops and 18 legumes; cereal production in 2016 was ca. 3000 Mt and legume production was ca. 500 Mt [22]. There are 13 genera (in six legume tribes) that constitute major legume crops [23,24]. Pea (*Pisum sativum* L.) belongs to the tribe Fabeae [25], which contains: *Lathyrus* (grass pea); *Lens* (lentils); *Pisum* (peas), *Vicia* (vetches) and monotypic *Vavilovia*. Although Schaefer et al. [25] showed that *Pisum* and *Vavilovia* are imbedded within *Lathyrus*, here we keep the Linnean designation *Pisum sativum* L. For *Pisum*, many species and subspecies names have been used [26,27], however most commonly, two species, *Pisum fulvum* Sibth. & Sm. and *P. sativum* L. are recognized. The latter is divided into two subspecies, the domesticated pea *P. sativum* subsp. *sativum* and the wild form, *P. sativum* subsp. *elatius* (M. Bieb.) Asch. & Graebn [28,29]. Geographically, *P. sativum* subsp. *elatius* is distributed widely across the Mediterranean basin from Spain to the Middle East and north to Central Europe, the Caucasus and the Caspian Sea, whereas *P. fulvum* is found around its eastern edge (Syria, Lebanon, Israel, Palestine and Jordan) [29,30]. *P. fulvum* forms a distinct clade in all molecular diversity analyses and is the only good candidate in the genus for a distinct species [30,31]. The taxonomic status of the Ethiopian pea has been discussed extensively and has varied from subspecies (*P. sativum* subsp. *abyssinicum* (A.Br.) Berger) to species (*Pisum abyssinicum* A.Br.) [23,28,29,32,33,34]. Molecular analysis has proposed that *P. abyssinicum* is not a subgroup of cultivated *P. sativum,* however that it has probably been domesticated independently from a separate *Pisum* genepool [31,33,34,35,36,37,38]. Taken together, the genus *Pisum* is best described as “a species complex with multiple sub-species which interbreed to different degrees” [31,33,34].

Both morphological and genetic studies have identified *P. sativum* subsp. *elatius* and *P. fulvum* as wild germplasm in that they have dehiscent pods and seed dormancy (thick testa), occur in the wild and are undesirable as a domesticated annual crop. In contrast, *P. sativum* subsp. *sativum* (including varieties *arvense, transcaucasicum* and *asiaticum*) are diagnosed by characters that are selected during domestication, namely: non-dehiscing pods, absence of seed dormancy and seeds with a smooth, thin testa [2,39]. The Ethiopian pea shares these domestication characteristics and they are the main reason why it was previously considered to be a sub-group of *P. sativum*.

Archaeological evidence dates the origin of cultivated pea 10,000 BC in the Near East [12,40] and Central Asia [10,41]. The archaeological evidence further suggests that the cultivation of pea spread from the Fertile Crescent westwards through the Danube valley, ancient Greece and Rome into Europe. Linguistic evidence additionally backs this suggestion [42]. During this same period, pea also moved eastward to Persia (now Iran and Afghanistan), India and China [27,43]. This might explain the novel diversity of Afghan type and Chinese landrace peas [29,44,45], either through genetic drift or through natural selection in diverse environments [46]. Similarly, human selection for early flowering as a drought avoidance phenotype may have acted on the Ethiopian pea (*P. abyssinicum*).

An independent domestication of the Ethiopian (*P. abyssinicum*) pea has been proposed by several authors [31,32,33,35,36,37] and a chromosomal translocation [47,48] that differentiates *P. sativum* and *P. abyssinicum* karyotypes is consistent with a distinct origin. Wild peas have not been described in Ethiopia, suggesting that it is unlikely to be a native plant. It was most likely introduced to Ethiopia along with barley [33,37,49]. *P. abyssinicum* has been reported in both Ethiopia and Yemen [33]. Human population genetic analyses have suggested that there are southern Arabia (Yemen) and Ethiopia blend African and Eurasian lineages [50]. The major episodes in the peopling of Arabia took place from north to south in the Late Glacial Maximum and, to a lesser extent, the immediate post-glacial/Neolithic ages [50]. Genetic connection between contemporary Ethiopians and Anatolian people, as well as archaeological evidence, dates the arrival of Near Eastern crop domesticates to the same time period as this late phase of the human history of Ethiopia (circa 3000 years ago). This suggests that the direct descendants of the farmers that earlier brought agriculture into Europe may have also played a role in the introduction of these crops to the Horn of Africa [51].

Pea genetic diversity that is conserved in genebanks has been extensively studied over the past two decades (reviewed in [52,53]). There are 25 large collections preserving pea diversity, together holding around 72,000 accessions. A further 27,000 accessions are distributed over 146 collections worldwide [23,29,52]. However, few (less than 1%) of these are wild pea relatives [52]. Molecular analysis of pea diversity preserved in germplasm collections has been carried out using various methods [30,31,33,35,54,55,56,57,58,59,60]. Recently, genotyping-by-sequencing was applied [61] to a set of 431 *P. sativum* (*sensu lato*) accessions, including 11 *P. sativum* subsp. *elatius,* 2 *P. abyssinicum* and 25 *P. fulvum* accessions, and a 13k singles nucleotide polymorphism (SNP) panel of mapped genes to 917 accessions, including 50 wild peas [55]. The largest number of accessions analyzed so far (3020 and 4200) were dominated by cultivated types and had relatively few (45) markers (retrotransposon-based insertion polymorphisms [29,62]). Genome-wide next generation sequencing techniques have been used recently to study the diversity of wild pea [30], such as a study that comprised of 143 *P. elatius* and 18 *P. fulvum* accessions. All of these studies indicated that *Pisum* is very diverse and that the diversity is structured, showing a range of degrees of relatedness that partially reflect taxonomic identifiers, eco-geography and, in the case of cultivated material, breeding gene pools.

In contrast with other well studied crops such as rice [63], barley [64,65], wheat [66,67], maize [68], chickpea [69], groundnut [70], common bean [71] and soybean [72], the domestication scenario in pea is not well known. In order to fill gaps in our understanding of pea domestication, we asked the following questions: (1) What is the relationship between wild *P. elatius* and cultivated *P. sativum* and *P. abyssinicum*? (2) Was *P. abyssinicum* derived from domesticated *P. sativum* or was it domesticated independently? (3) Might *P. abyssinicum* originate from hybridization between *P. fulvum* and *P. elatius*?

## 2. Materials and Methods

### 2.1. Plant Material

*Pisum* taxonomy has frequently been revised without adding much clarity as to the partitioning of genetic variation within the genus. Here we adopted a scheme that treats the genus as monospecific and accepts the Linnean term *Pisum sativum*, while acknowledging that Schaefer et al. [25] clearly demonstrated that *Pisum* is embedded within *Lathyrus* (and *Lathyrus* within *Vicia*). For simplicity, we omitted the intermediate level species name because it is common to all taxa, thus, *Pisum sativum* corresponds to *Pisum sativum* subsp. *sativum*, *P. elatius* to *Pisum sativum* subsp. *elatius*. For cultivated pea, we used *Pisum sativum* L. as described by Linnaeus [73]; *Pisum abyssinicum* A.Braun [74] was used for the Ethiopian form of cultivated pea. For wild pea, we used *Pisum elatius* M. Bieb. [75] in the broad sense and *Pisum fulvum* Sibth. & Sm. [76].

A panel consisting of 154 wild *P. elatius* (134) and *P. fulvum* (20) from Smýkal et al. [30,77] and 74 domesticated (64 *P. sativum* landraces and 10 *P. abyssinicum*) accessions were sampled to maximize the geographic diversity of the wild and landrace pea habitats (Table S1). The set of *P. sativum* landraces (64) originated from several geographical regions: Middle East (Turkey, Syria, Cyprus, Lebanon) 12, Caucasus region (Georgia, Russia, Armenia) 13, Europe (Greece, Balkan, Italy, Spain and Central Europe) 10, Central Asia (Afghanistan, Iran, Pakistan, Tajikistan, Nepal) 10, Ethiopia and Northern Africa (Morocco, Libya) 10, China and India (10). Sampling was based on our previous studies [29,31]. The material originated from several major genebank collections (Table S1) and was largely of single-seed descent. In terms of wild (*P. elatius*) accessions, 134 covered the geographic range of the species [30]. Of these 90, they were from the Middle Eastern center (Turkey, Syria, Israel, Jordan), 33 were from Europe (Western, Eastern Mediterranean, Hungary, Italy), 8 were from the Caucasus (Russia, Armenia, Georgia) and 3 were from Northern Africa (Algeria, Morocco). Twenty *P. fulvum* were sampled: Israel (6), Syria (12), Jordan (1) and Turkey (1). All ten *P. abyssinicum* accessions were from Ethiopia. The latitude and longitude for the collection points of the wild pea samples were obtained from germplasm passport data and were processed using ArcGIS for Desktop (version 10.4, http://desktop.arcgis.com). All of the wild material was purified by single-seed descent for several generations to eliminate accession heterogeneity. Leaf samples were taken from a single plant per accession and were freeze dried before DNA isolation.

### 2.2. DArTseq Analysis

Genomic DNA was isolated from approximately 100 mg of dry leaf material using the Invisorb Plant Genomic DNA Isolation kit (Stratec Molecular, Berlin, Germany). Standard diversity array technology sequencing (DArTseq) analysis [30,77] was undertaken at Diversity Arrays Technology Ltd. (Canberra, Australia) using proprietary methodology [78,79,80].

In brief, DNA samples were processed in digestion/ligation reactions [30,77], however a single *PstI*-compatible adaptor was replaced with two adaptors. The *PstI*-compatible adapter was designed to include Illumina flowcell attachment sequence, sequencing primer sequence and barcode region. The reverse adapter contained flowcell attachment region and *MseI*-compatible sequence. Only mixed fragments (*PstI-MseI*) were effectively amplified in 30 rounds of PCR using the following reaction conditions: 94 °C for 1 min, 30 cycles of: 94 °C for 20 s, 58 °C for 30 s, 72 °C for 45 s and a final extension of 72 °C for 7 min. Afterwards, PCR equimolar amounts of amplification products from each sample were bulked and sequenced on Illumina Hiseq2500 (San Diego, CA, USA) which was run for 77 cycles.

### 2.3. Bioinformatic Data Processing

DArTseq analysis of 228 pea samples, each with 75,862 DArTseq fragments, yielded 0.98, 2.19, 1.24 and 0.02 million scores for -, 0, 1 and 2, respectively. The “-” are missing data (22%), “0” and “1” are alternative sequences for a given fragment and “2” indicates that both the “0” and “1” sequences were detected. Taking into account the single seed descent of most of the material and based on our previous experience using the same DARTseq procedure on a recombinant inbred lines (RIL) mapping population (F_6_) and wild-collected material [75], we concluded that “2” (0.45% of total markers) most likely represents sequencing error or paralogous sequences. Accordingly, both “-” and “2” scores were treated as missing data.

These 75,862 DARTseq fragments were filtered to maximum 20% missing data (Table S2). We used 11,343 polymorphic high-quality SNPs (HQ SNPs), with one SNP per locus mapped to a pea genome assembly (BioProject: PRJNA432052, GenBank assembly: GCA_003013575.1), thereby avoiding an uneven representation of markers by genomic location.

#### 2.3.1. Genetic Structure and Diversity Analysis

To describe diversity and differentiation, we used a series of complimentary analyses to examine the relationships among accessions in a step-wise fashion beginning with ordination analyses, which have few assumptions, and progressing to model based admixture analyses. We first used Principal Coordinate-Model Clustering (PCO-MC), a principal coordinates based density clustering procedure, to identify distinct genotypic clusters [81]. To perform the ordination, a simple band sharing coefficient was used to calculate the necessary genetic distance matrix from homozygous co-dominant DArTseq data [81]. We then constructed a reticulate network using the NeighborNet algorithm [82] that was implemented in SplitsTree 4.14.4 [83]. Phylogenetic networks are generalizations of phylogenetic trees that represent conflicting signals in data derived from non-tree like processes, such as hybridization followed by introgression. The network displays relative evolutionary distances between taxa as well as uncertainty in the groupings in the form of “splits” (or “webbing”) of internal branches. In addition, we applied a discriminant analysis of principle components (DAPC) that was implemented in the adegenet package in R [84] for visual exploration of the data. The number of clusters for the DAPC analysis was set to 10, according to the value of Bayesian Information Criterion (BIC). DAPC clustering was performed using five Principal Component Analysis (PCA) axes and three Discriminant Analysis (DA) axes. Finally, a coancestry matrix among accessions was inferred using the Markov Chain Monte Carlo (MCMC) clustering algorithm of RADpainter and fineRADstructure v0.3.2 [85] which has been developed for large SNP datasets (specifically, RADseq). The fineRADstructure analysis used 500,000 burn in steps and 500,000 sample iterations to infer population structure.

#### 2.3.2. Seed Phenotyping

Seeds were harvested from 3–5 plants per pot and accession was grown in five litre pots with peat-sand (90:10) Florcom Profi substrate mix (BB Com Ltd., Letohrad, Czech Republic) in glasshouse conditions (January–May 2016 and 2017) and a natural photoperiod (Palacky University campus, Olomouc, Czech Republic). After harvest, mature seeds were cleaned from the pods, were dried at room temperature and were packed in paper bags. Germination of intact seeds was tested at 25/15 °C in the dark at 14/10 h (day/night) on water saturated filter papers (Whatman Grade 1, Sigma, Prague, Czech Republic) in 90 mm Petri dishes (P-Lab, Prague, Czech Republic) in temperature-controlled chambers (Laboratory Incubator ST4, BioTech, Prague, Czech Republic). Then, 25 seeds per accession were incubated in duplicate. Seeds were monitored at 24 h intervals for a total of 28 days. Seeds were considered germinated when the radicle protruded from the testa. The cumulative percentage of germinated seeds was calculated [86]. Samples were classified as “dormant” if by the end of the test period (28 days), fewer than 50% of the seeds had germinated and, conversely, were classified “non-dormant” if over 80% had germinated. Seed batches with 50–80% germination were classified as intermediate. Photographs of ten individual seeds per genotype were taken with an Olympus SZ61 stereomicroscope (Olympus Corp., Tokyo, Japan) that was equipped with an Olympus E-410 digital camera (Olympus Corp., Tokyo, Japan). The photographs were processed by QuickPHOTO MICRO 3.0, which was supplemented by the Deep Focus 3.3 module (PROMICRA, Prague, Czech Republic). Accordingly, the seed coat was classified either as smooth or rough and either as pigmented or not pigmented [87].

## 3. Results

### 3.1. Diversity Array Technology Analysis and Bioinformatic Processing

**Figure 1:**
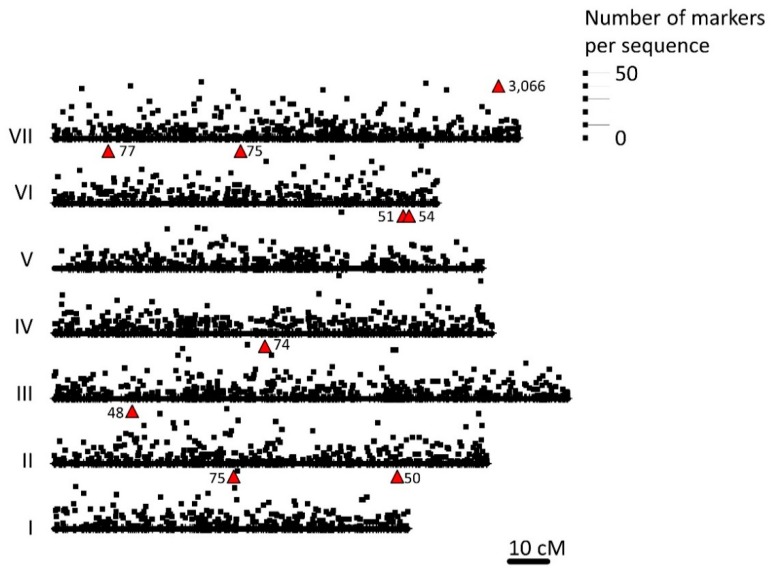
The distribution of 1929 loci, corresponding to 3838 different sequences, mapped by Tayeh et al. [57] that match the diversity array technology (DArT) sequences scored in this study is presented on the *x* axis for each pea linkage group (I to VII). The *y* axis gives the number of DArTseq markers corresponding to each of these sequences corresponding to the scale on the right. Where the number of sequences would be off scale or obscured by those of another linkage group, the number is indicated adjacent to a red triangle.

A total of 75,862 different sequences were obtained by DArTseq; of these, 66,910 were polymorphic. Further, 72,679 (95.9%) sequenced fragments could be mapped to a shotgun pea genome (GCA_003013575.1) and, of these, 22,013 (61.9%) could be mapped to the pea RNA atlas; these correspond to 8405 unique sequences on the pea Infinium BeadChip (Illumina, SanDiego, CA, USA), known as the Genopea 13.2K SNP Array. These Infinium BeadChip sequences corresponded to 3838 positions on the genetic map of Tayeh et al. [55] which were distributed across all the linkage groups. The distribution of these loci on the pea linkage groups is shown in Supplementary Table S2 and Figure S1. After filtering according to missing data and minor allele frequency cutoffs (see Materials and Methods), 11,343 SNPs remained for analysis (Figure 1).

### 3.2. Genetic Structure and Diversity Analysis

**Figure 2:**
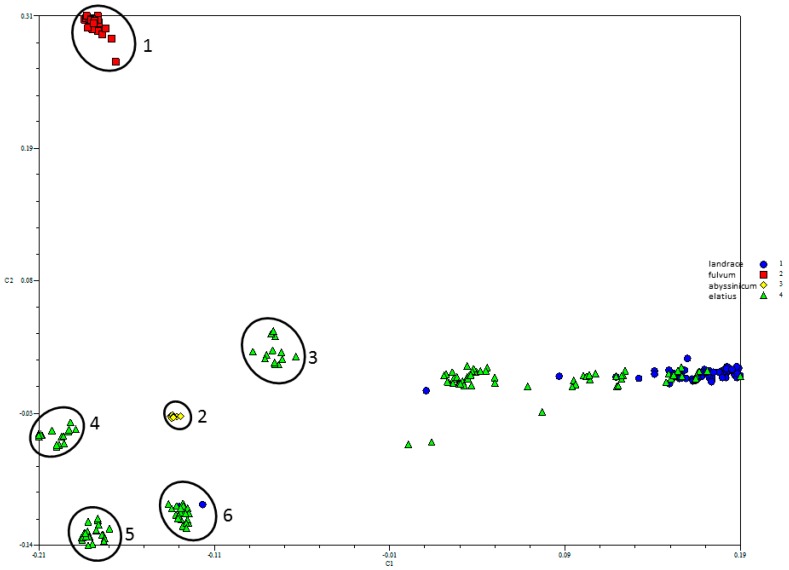
Six distinct genetic clusters identified using Principal Coordinate-Model Clustering (PCO-MC), a principal coordinates-based clustering procedure. Clusters 1 and 2 contain exclusively *P. fulvum* (red squares) and *P. abyssinicum* (yellow circles), respectively. The remainder contains distinct segregates of *Pisum elatius*, with the exception of cluster 6 which also includes *P. sativum* landrace accessions TG2548 and PI343972. An intergrading cloud of *P. elatius* (green triangles) and *P. sativum* landrace (in blue) material remained unassigned.

The PCO-MC analysis identified 6 distinct clusters within the SNP data (Figure 2). Both *P. fulvum* (cluster 1, red squares) and *P. abyssinicum* (cluster 2, yellow diamonds) formed distinct groups with high stability values, 96% and 71%, respectively. With this method, stability values > 15% generally mark true positive genetic clusters. Additionally, a fraction of the *P. elatius* accessions (in green) cluster into four distinct groups (3–6 in Figure 2). Group 3 comprised of 11 *P. elatius* accessions from eastern Turkey (T14-2, T14-9, T15-1, T15-5, T15-11, W6-26109, W6-26127, JI261, P013, PI344001, PI344002) and two from Israel (711, PI560059), group 4 comprised of 16 *P. elatius* accessions largely from Israel (7), Jordan (1) and Turkey (3), however three from Italy, Spain and Algeria, group 5 comprised of 22 *P. elatius* accessions largely of European origin and, finally, group 6 comprised of 20 *P. elatius*, however also two *P. sativum* (PI343972, TG2548) accessions. The remaining 63 *P. elatius* (green) and 62 *P. sativum* accessions (in blue) form a continuum with accessions that were interspersed with one another in the PCO bi-plot.

**Figure 3:**
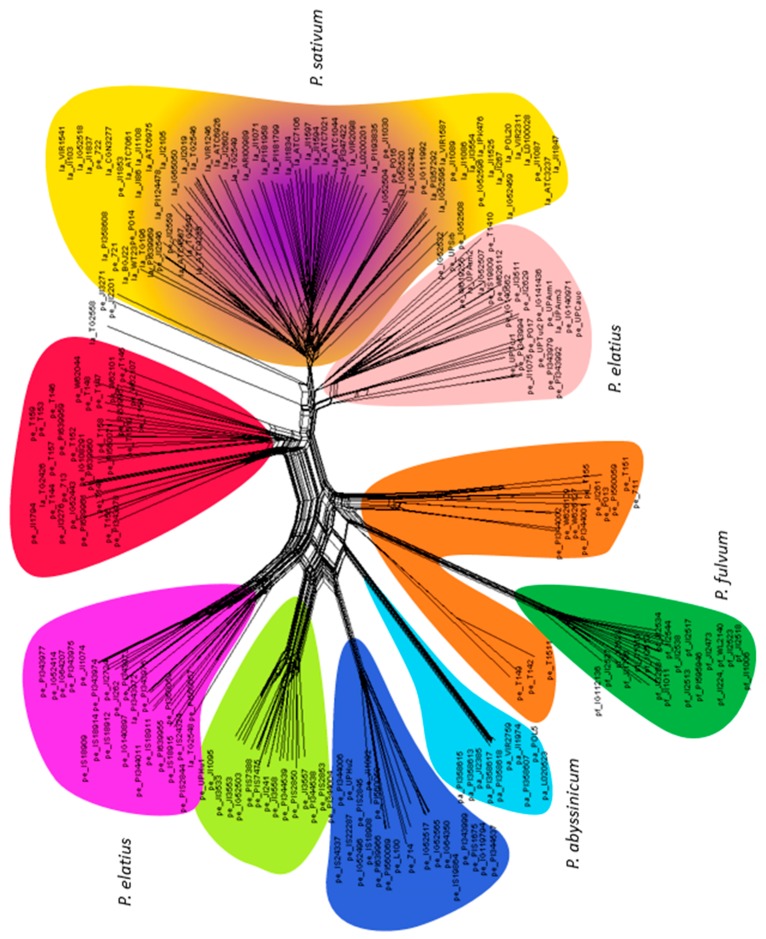
NeighborNet network shows reticulate genetic relationship between pea accessions. Color coding corresponds to the discriminant analysis of principle components (DAPC) K10 clusters of Figure 4.

**Figure 4:**
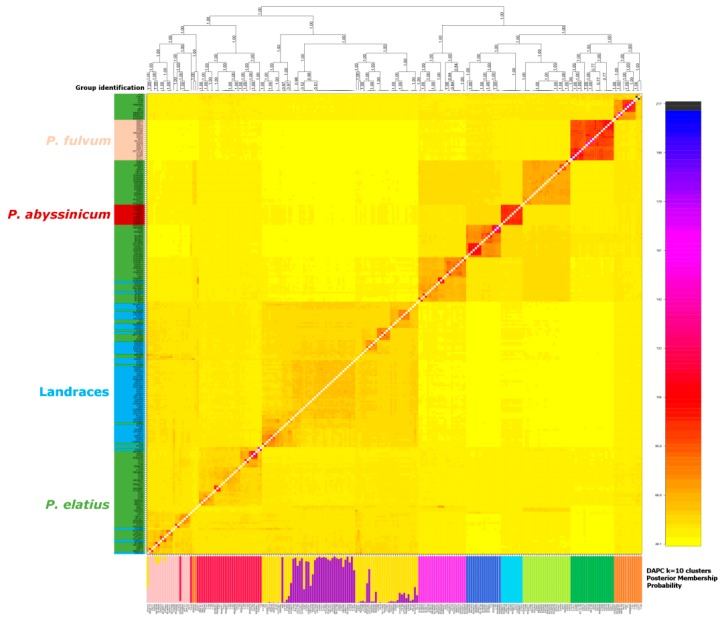
Coancestry matrix for 228 individuals based on HQ SNPs (high-quality single nucleotide polymorphism) dataset calculated by fineRADstructure. The heat map depicts the high resolution genetic relationship structure of individuals selected across *Pisum*. The lower bar plot shows the results of the DAPC analysis of 10 identified groups.

NeighborNet was used to construct a phylogenetic network (Figure 3). The narrow reticulation leading to *P. abyssinicum* showed its high genetic uniformity and its broad base indicated its affinity with two *P. elatius* groups (colored dark blue and brown) as well as *P. fulvum* (in dark green). A group of *P. fulvum* was well separated with an internal bifurcation. There were seven separate groups of *P. elatius,* in agreement with DAPC analysis (Figure 4). Four of them correspond to PCO-MC groups 3 to 6 (Figure 2), while NeighborNet analysis further separated 3 additional groups of *P. elatius,* one overlapping with *P. sativum* group. Domesticated *P. sativum* landraces have a complex reticulate structure and are not separated from the central reticulation by a long branch.

DAPC identified 10 clusters of accessions that were designated Group 1 to Group 10 in Figure 4. Group 1 (red) contained 30 accessions, all except for three from Turkey. All Group 2 (dark green) accessions were *P. fulvum* and all *P. fulvum* accessions were assigned to this group. Group 3 (yellow) was a mixture of *P. elatius* and landraces of *P. sativum*. Group 4 (dark blue) comprised *P. elatius* accessions, seven from Israel, six from south-eastern Turkey and one each from Jordan, Algeria and Italy. Group 5 (brown) comprised 12 accessions from eastern Turkey and two (711, PI560059) from Israel. Group 6 (purple) comprised seven *P. elatius* accessions from Georgia, two from Armenia one from southern Turkey, and one from Ethiopia. Group 7 (light blue) comprised all 10 *P. abyssinicum* accessions. Group 8 (magenta) comprised 19 accessions, all except for one (PI560057, from Portugal by passport data assigned as *P. sativum* landrace) that was of Middle East origin. Group 9 (light green) comprised 22 accessions of various geographical origins: Western and Central Mediterranean (nine), Eastern and Southern Europe (eight) and Middle East (four). Group 10 (pink) contained 16 *P. elatius* and two *P. sativum* (TG2568, UP-Arm3) accessions. Groups 4, 8, 9 and 10 correspond to PCO-MC identified groups 3 to 6, which are clearly separated.

fineRADstructure described the shared ancestry between accessions (Figure 4). The analysis shows blocks of related accessions as orange to red squares, and these have a nested structure. *P. abyssinicum* and *P. fulvum* are seen as distinct and relatively homogenous groups. It showed two large groups, one including *P. fulvum*, *P. abyssinicum* and four sub-groups of *P. elatius* accessions, corresponding to DAPC groups 1–4 and to group 4 which was identified by PCO-MC. The second large group contained two subgroups of *P. sativum* landraces and two subgroups of *P. elatius* accessions. In this case, the internal structure was noticeably graded. FineRADstructure analysis identified a group of 11 *P. elatius* accessions from eastern Turkey (T14-2, T14-9, T15-1, T15-5, T15-11, W6-26109, W6-26127, JI261, P013, PI344001, PI344002) and two from Israel (711, PI560059) which are ancestral to *P. elatius* and *P. fulvum*. There was an overall good correspondence between the groups as inferred from DAPC, the distance tree and from fineRADstructure (Figure 3 and Figure 4).

### 3.3. Seed Phenotyping

Seed dormancy, caused by a water impermeable seed coat, is typical of wild legume species, including wild pea. This trait has been lost during domestication. We analyzed mature dry seeds for seed coat pigmentation and surface properties. Wild pea seeds are typically rough (as, for example, determined by the gene *Gritty*), while seeds of cultivated pea usually have a smooth and thin testa. All 64 *P. sativum* landraces had non-dormant seeds (Table S1), germinating with the fraction 0.8 ± 0.057 germinating within 48 h of being exposed to water, and all except four (PI343972, IG52507 from Turkey and UP_Arm2, UP_Arm3 from Armenia) had a smooth testa (*gritty*). Forty accessions had a pigmented seed coat, while 24 did not. *P. abyssinicum* seeds were smooth, sometimes glossy, and were either pigmented or not. Importantly, these were also non-dormant and imbibed readily. Seeds of *P. fulvum* were all dormant and pigmented (usually black or dark brown). The seed coats of *P. elatius* accessions were mostly pigmented and rough as well as dormant (103 out of 134). Further, 15 accessions had smooth, pigmented testa—four of which were dormant, while 11 were non-dormant. Three accessions of *P. elatius* (JI1030, IG111992, JI1853) had a smooth testa, were non-pigmented and non-dormant—a combination that could be taken as indicative of a cultivated type. Two accessions (JI1075 from Turkey and JI2201 from Russia) had a rough testa, were non-pigmented and non-dormant (Table S1).

## 4. Discussion

Here, we have undertaken a comprehensive study of wild *Pisum* together with landrace material. This work gives a more comprehensive view of pea genetic diversity in relation to domestication than previous studies [29,30,31,61] by combining high density, genome wide, marker assays with a targeted set of accessions.

### 4.1. Pisum Taxonomy

Phylogenetic studies have layered new complexities in the classification of *Pisum*. Analysis using chloroplast genes nested a monophyletic *Vicieae* tribe within *Trifolieae*, with the genus *Trifolium* as sister to the *Vicieae* [25,88]. The genus *Vicia* was shown to be paraphyletic, with the genera *Pisum, Vavilovia, Lathyrus* and *Lens* nested within *Vicia*. Schaefer et al. [25] proposed that one solution to resolve the paraphyly of *Vicia* would include transferring *Pisum* and *Vavilovia* to *Lathyrus*. In their analysis, *Pisum* and *Vavilovia* were each monophyletic and together monophyletic within *Lathyrus,* noting that *Vavilovia* had previously been included within *Pisum*. If this scheme were to be accepted, then *Vicia* section Ervum must also be elevated to the status of a genus and the precedent would be to rename *Pisum* L. as *Lathyrus oleraceus* Lam. [89]. This classification has already been used [90] in accordance with the ‘International Code of Nomenclature of algae, fungi and plants’ (International Code, 2012). Subsequently, Kosterin [91] has renamed *Pisum sativum* as *Lathyrus schaeferi* Kosterin nomen novum pro *Pisum abyssinicum* A. Braun and *Lathyrus fulvus* (Sibthrop et Smith) Kosterin combinatio nova pro *Pisum fulvum* Sibthrop et Smith.

For the purpose of our study, we have retained the commonly understood *Pisum* L., as indicated in the Materials and Methods section, and in the absence of agreement on the wholesale reclassification of these taxa. As a direct result of its broad phenotypic diversity, a large number of different Latin names at different ranks have been proposed for various forms of pea [29,37]. The classification of *Pisum* L. based on morphology and karyology clearly delineates two species, *P. fulvum* Sibth. & Sm. and *P. sativum* L. [92,93]. *P. sativum* has been further divided into three taxa recognized either as subspecies or species: *P. elatius* Bieb. [75], *P. humile* Boiss. & Noe and *P. syriacum* Boiss. & Noe. [27]. *P. elatius* M. Bieb. was first described at the rank of species in 1808 [75] and was later reduced in rank to a subspecies [94], although many authors ascribe this to Ascheron and Graebner [95]. *Pisum humile* was described by Boissier and Noë [96], however their name was illegitimate because it is a later homonym of *P. humile* Miller [97], a form of cultivated pea. Berger [98] downgraded that to a subspecies and gave it a new name: *P. sativum* subsp. *syriacum* A. Berger, however its status was raised again to species by Lehmann [99] as *P. syriacum* (A. Berger). C.O. Lehm., though this nomenclatural change remained unsupported. The work of Ben-Ze’ev and Zohary [47] has become the standard text for pea species relationships and was based on classical species definitions using hybridization barriers along with ecological aspects of distribution. Their work followed the taxonomy of Boissier [96], which recognized three wild pea species: *P. fulvum, P. sativum* subsp. *elatius* Bieb. and *P. humile* Boiss & Noë (=*P. syriacum* (A. Berger) C.O. Lehm.), and the domesticated pea *P. sativum*. These two wild groups of *P. sativum* subsp. *elatius* Bieb. and *P. humile* Boiss & Noë (=*P. syriacum* (A. Berger) C.O. Lehm.) were described as being morphologically, ecologically and genetically distinct [25,45]. Recently, Ladizinsky and Abbo [100] also recognized two groups: subsp. *elatius* and subsp. *humile* and further described two varieties of subsp. *humile*, a “southern” and a “northern” form, based on geographic distribution. They delimited the southern form as subsp. *humile* var. *humile* (Boiss et Noë) Ladizinsky, and the northern form as subsp. *humile* var. *syriacum* (A. Berger) Ladizinsky. Recent comprehensive genome wide analysis of 150 *P. elatius* and *P. fulvum* samples [30], however, did not support this geographical or morphological division, although *P. elatius* diversity was structured into five groups.

Based on the results of this study, we propose to use *Pisum sativum* L., as described by Linnaeus [73], for cultivated pea, *Pisum abyssinicum* A. Braun for the Ethiopian form of cultivated pea and for wild pea to retain *Pisum fulvum* Sibth. & Sm. and to use *Pisum elatius* M. Bieb. in the broader sense that includes the rest of the genus.

### 4.2. Pisum Genetic Diversity

The current study included the samples used by Smýkal et al. [30], however added *P. abyssinicum* and *P. sativum* cultivated material. It should be noted that the marker set was slightly different as this analysis included the combination of two DARTseq results which were newly computationally processed. This was likely one of the reasons why 47 out of 161 samples that were common in both studies were assigned to different genetic clusters (Table S1). The second reason could be that the addition (or removal) of any samples affected the analysis and the use of slightly different analytical methods.

In the PCO analysis (Figure 2), *P. fulvum* and *P. abyssinicum* were assigned to single distinct groups, while wild *P. elatius* formed four distinct groups plus residual, un-assigned samples that overlap with *P. sativum* landrace material, which is suggestive of a shared history between *P. sativum* and *P. elatius*. *P. abyssinicum* was clearly distinct from all *P. sativum*. There was good agreement in the grouping of *P. elatius* assignments between this and a prior study [30] in three of the *P. elatius* clusters (Table S1), while the remainder *P. elatius* were differently assigned.

**Figure 5:**
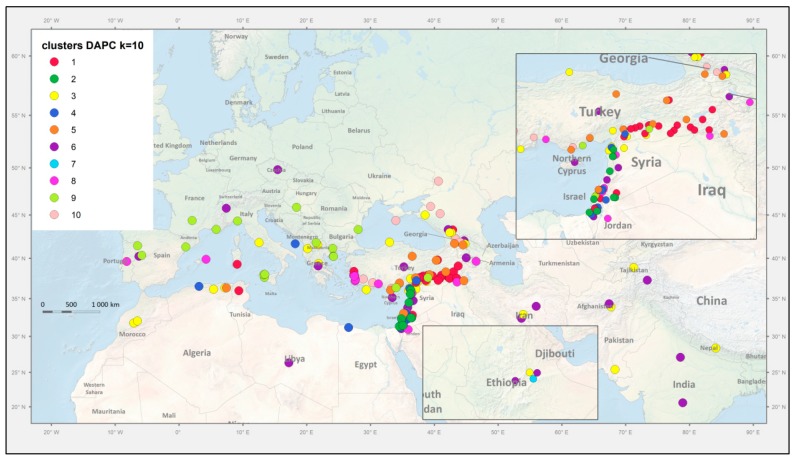
Geographic distribution of collection sites. Markers are coloured according to the assignment to respective genetic groups identified in Figure 4. *P. sativum* landraces (in green) are placed centrally in their respective country of origin.

We have previously [30] analyzed the spatial distribution of the geographical pattern to the genetic structure of wild *P. elatius* accessions by the centroid approach and found the clusters were mostly all overlapping without clear isolation by distance. In the Middle East, the center of origin of the Fabeae tribe origin [25], the genetic diversity of *Pisum* accessions, was greater than elsewhere [30]. In Europe, one sample from Portugal and one from the Balearic Islands were distinct from all other European *P. elatius* which belong to two groups, one with affinity to domesticated *P. sativum* and the other a distinct subgroup of *P. elatius* (Figure 4 and Figure 5). There was small (12 accessions) DAPC group 5 of *P. elatius*, which all except for one originated from eastern Turkey and had a significant proportion of *P. fulvum* alleles. This group corresponds to Q6 in our previous study [30]. This material, with the exception of JI261 [31], was not included in previous studies [55,56,61,62]. The complete plastid genome sequence of accession W6-26109 from this group 5 was recently shown to match *P. fulvum* [101]. It was suggested that this accession belongs to an ancestral group from which *P. elatius* and *P. fulvum* were derived. In this case, both the plastid [101] and nuclear [this study] genomes suggest the same conclusion and likely reflects a shared ancestry. The other accessions that group 5 (Figure 5, Table S1) were collected from was north-eastern Turkey outside the past and present [30] geographical distribution of *P. fulvum*. This is newly identified diversity in *Pisum* genus and indicates that there is potential for further collecting new diversity.

In our study, we used fewer cultivated accessions than Jing et al. [31], however the selected 64 landraces in this study came from a broad geographical area, yet they were grouped together. DAPC identified two groups of *P. sativum* accessions, one with 30 and the other with 27 accessions, however the groups were not geographically distinct. The remaining seven *P. sativum* accessions were incorporated into DAPC groups that mostly comprised *P. elatius*. These possibly correspond to mis-identification in the passport data (as mentioned in [62]), or *P. elatius-P. sativum* introgression (similar to introgression in chickpea, [102]), however this could also be the signature of additional domestication events. The accessions TG2548, TG2426, TG2558, from Georgia, have been described as morphologically distinct having a vetch-like appearance and were assigned to a subspecies: *P. sativum* subsp. *transcaucasicum* [26]. Except for TG2548, they have smooth seed coats and non-dormant seeds.

Our data showed no clear separation of subgroups within *P. sativum* in contrast with findings of Siol et al. [55] using an Illumina SNP assay. Siol et al. [55] observed three large genetically distinct clusters: (1) wild peas and landraces from the Middle East and Asia, (2) winter peas and (3) spring peas varieties; in addition to these three groups, these authors identified distinct groups from the Far East and China, as well as Central Asia (Afghanistan, Pakistan, Nepal). Similarly, using a genotyping by sequencing (GBS) based analysis [61] of 431 largely cultivated types, a separate group from Central Asia (defined as Afghanistan, China, India, Nepal and Pakistan) which were closer to *P. elatius* was identified.

### 4.3. Pea Domestication

The ever-intriguing question of crop domestication is whether it happened only once or multiple times. For pea, conclusions must be drawn from extant material as archeological samples cannot yet be examined at a comparable level of detail. In the case of domesticated plants, multiple origins might be masked by hybridization which may bring independently domesticated stocks together. In several crops, there is evidence of one (maize, [68], chickpea, [69], soybean, [72], groundnut, [70]) or two (common bean, [71]) domestication events. It was recently shown that multiple origins might be revealed as genome mosaics (barley, [64,65], rice, [63] and emmer wheat, [67]). Multiple domestications where gene flow occurred early in the process have also been reported [65,103]. In most of these studies, a strong genetic bottleneck was detected.

A description of domestication for several legume species is only beginning to emerge (chickpea [69], groundnut [70] or common bean [71] and soybean [72]). One of the best studied legume genera is common bean (*Phaseolus*), with *P. vulgaris* having two gene pools and two independent domestication events that contributed to the modern common bean crop (reviewed in [71]). On the other hand, while the two types of cultivated chickpea, *desi* and *kabuli*, display large genomic differences, they were derived from a single domestication event [104,105]. In soybean, a combination of archaeological and molecular data suggested that a prolonged period of low-intensity management or semi-cultivation of wild soybeans at multiple locations preceded domestication [72]. These pre-domesticates may have been assimilated within wild soybeans or were integrated into the domesticated soybean [72]. This might also be the case in pea, being first gathered from wild stands, then cultivated [106], although the morphological distinction between the wild and domesticated plants remains problematic. Reticulate evolution during the domestication of emmer wheat has been proposed [67].

Our data showing overlap between domesticated and wild pea suggests a similar scenario. In contrast to wild cereals which occur in large stands [64,65,66], wild peas occur in scattered patches within which the populations are likely closely related [77]. During the pre-domestication period, it is possible that pea seeds were collected over a large area and were brought to a common point, likely creating mixed stands. In spite of open-pollination being rare in pea [77], in mixed stands, considerable diversity may remain during the domestication process due to sporadic gene-flow between lineages.

It has been argued that seed dormancy would have been a substantial barrier to the efficient cultivation of legumes during their domestication process, so it has been proposed that non-dormant types existed in wild populations that were selected by humans [11]. Results from the experimental cultivation of wild peas suggest that the crucial trait in pea domestication was the loss of the seed dormancy [107]. It has been proposed that at early stages of domestication, selection acted on standing variation, with further refinement of specific traits narrowed during and post-domestication [2]. Until now, we have had very little information on domestication genes in legumes (reviewed in [108]). Weeden [109] suggested that at least 15 genes are critical for pea domestication and our recent study of pod dehiscence and seed dormancy identified several candidates for these traits [87]. Here, we identified a group of (wild) *P. elatius* from Armenia, Georgia and south-eastern Turkey that are the most closely related wild peas to the cultivated *P. sativum* genepool (Figure 3, Table S1). Either these are genetically the closest extant progenitors of domesticated pea, or they represent early escapes from cultivation with a reversion to the wild type. Wang et al. [110] showed that analyses including “feral” rice i.e., wild rice that carries a causative domestication allele [111] alters the way wild rice and domesticated rice accessions are clustered [110]. Interestingly, feral origin for *P. sativum* subsp. *humile* var. *humile* was suggested [112]. However, Ben-Ze´ev and Zohary [47] noted that subsp. *humile* differs by one reciprocal chromosomal translocation from domesticated pea, which is not consistent with the claim of Abbo et al. [112]. Ladizinsky and Abbo [100] later proposed that pea domestication involved more than one cytotype. Jing et al. [31] and Siol et al. [55] proposed that the high genetic diversity in cultivated pea indicates a relatively weak domestication bottleneck and that genetic diversity may have been maintained because of the diversity of uses and the wide range of environmental conditions in which it is grown. Alternatively, this might indicate either multiple domestication events and/or prolonged wild to crop gene flow as has been shown in barley [65], emmer wheat [67] and chickpea [102]. Even today, in many areas of Middle East dryland, cropping is carried out in small patches of open woodland, which very often is in close vicinity to wild crop progenitors (Smykal, Berger, *personal observations*), thus enabling gene flow in both directions. This could contribute to the observed admixture both in wild *P. elatius* and cultivated *P. sativum* landraces groups. The Central Asian peas, sometimes called Afghan types or *P. sativum* subsp. *asiaticum* (Govorov) as defined by Govorov [26] and Makasheva [27], was discussed by Jing et al. [31] who supported the proposal of Young and Matthews [113] that this group is not solely characterized by the nodulation phenotype. In our analysis, these were represented by eight accessions (CGN3277, IPK476, JI86, JI103, JI2019, PI124478, VIR1246, PI639969) all of which, except for IPK476 and VIR1246, formed a distinct branch in SplitsTree (Figure 3). This is in agreement with previous studies [31,55,61] showing distinction of this group. In contrast, Chinese origin landraces, which were stated to be distinct [45], were distributed among several different branches within the group that contained *P. sativum* (Figure 3 and Figure 4, Table S1).

### 4.4. Independent Domestication of the Ethiopian (Pisum abyssinicum) Pea

*P. abyssinicum* was first described [74] at a species rank (the type specimen was collected in 1840 by Schimper W.H and is stored at the herbarium of National Botanic Garden of Belgium (BR, BR0000006255831) and this was adopted by many authors [26,28,29,31,33,34], while others considered it a subspecies of the common pea *P. sativum* subsp. *abyssinicum* (A. Br.) Berger [27,98], or only a form [48,110,114]. Ethiopian pea (*P. abyssinicum*) called Dekoko (‘minute seeded’, in Amharic) is considered endemic to Ethiopia and southern Yemen. It was first described in Tigray and Amhara regions in northern Ethiopia. According to a report [115], it used to be more common and, currently, its cultivation is restricted to South Tigray and North Wollo provinces and Southern Yemen where it accompanies the main cereal crops, chickpea, linseed and grasspea. Common pea (*P. sativum*) is also grown in Ethiopia, however the Ethiopian pea (*P. abyssinicum*) is valued for its higher nutritional quality [115].

The intriguing question is: was Ethiopian pea domesticated independently? And if so, was it domesticated in place or brought from the Middle East? An independent domestication of the Ethiopian pea (*P. abyssinicum*) has been proposed by several authors [31,32,33,34,36,37] and is supported by it having a distinct karyotype [47,48,114]. The *P. abyssinicum* genome is reportedly slightly (ca. 8%) larger than that of *P. sativum* [116]. Serological studies of *Pisum* taxa [117] suggested that *P. abyssinicum* might have originated from hybridization between *P. sativum* subsp. *elatius* and *P. fulvum*. Retrotransposon based diversity analysis showed substantial marker sharing between both [33,34]. Similarly, an extended study [31] showed that *P. abyssinicum* shares several phenotypic traits and a significant proportion of molecular marker alleles with *P. fulvum* and tends to occupy an intermediate position between the latter and *P. elatius* in molecular diversity plots, which is consistent with the possibility that progenitor of *P. abyssinicum* shared some ancestry with what is now *P. fulvum* and also with *P. elatius*. Since *P. fulvum* and *P. elatius* are sympatric in Israel [100], it might be that a naturally occurring hybrid was recognized by humans and was moved to Ethiopia and southern Arabia. Although wild pea is unlikely to be native to Ethiopia, the possibility of wild pea species having occurred in North Africa is supported by an intriguing record of *P. elatius* seeds (JI254) from a market in Ethiopia [118]. *P. abyssinicum* might have its origin within its present range, which is itself a known center of origin for cultivated plants [15]. The distribution of wild *Pisum* species may have also changed since the time of domestication. The desertification of the Sahara is known to have occurred in two abrupt phases, the last of these possibly as recent as 3500BP [119]. However, it seems most likely that domesticated *P. abyssinicum* was introduced via human migration [51]. The earliest known remains of *P. abyssinicum* (from present day Eritrea) are dated back to approximately 400AD [32] and are considerably more recent than the archaeological finds of *P. sativum*. Our study confirms prior studies suggesting that *P. abyssinicum* is a distinct genetic lineage [31,32,33,34,120]. The very low genetic diversity present in *P. abyssinicum* is the result of a severe genetic bottleneck and its allelic composition suggests rather ancient divergence of *P. abyssinicum* from other *Pisum* lineages. It is likely that specific allele composition contributes to the narrow eco-geographical range of *P. abyssinicum*. Although both species are commonly grown in Ethiopia, a reproductive barrier prevented gene flow between them [47,91,121]. Recently, the issue of the Ethiopian pea was revisited [38] using 54 gene sequences on a set of 76 cultivated pea (*P. sativum* subsp. *sativum*), two wild pea (*P. sativum* subsp. *elatius*), 11 *P. fulvum* and one *P.s.* subsp. *abyssinicum* sample, which demonstrated a close relationship among the three *P. sativum* subspecies and rejected the hypothesis that *P.s.* subsp. *abyssinicum* was formed by hybridization between one of the *P. sativum* subspecies and *P. fulvum*. This study [38] supported its status as a distinct subspecies, *P. sativum* subsp. *abyssinicum*. Crosses between *P. abyssinicum* and cultivated *P. sativum* showed some segregation of pod dehiscence, a domestication trait, suggesting that this might be governed by different genes in the two groups [118]. These populations will provide valuable tools to test the domestication scenario further once the respective domestication genes are identified. Identification of the genes underlying key domestication traits in pea [87,108] as well as the availability of the pea genome sequence should shed light on the number of domestication events in pea.

## 5. Conclusions

The results indicate that *P. abyssinicum* and *P. sativum* were derived from different genepools, thus representing two independent domestication events. The data does not support the hypothesis of a hybrid origin of *P. abyssinicum* nor of it being derived from *P. sativum*. *P. fulvum* is genetically distinct, while *P. elatius* diversity is structured to seven identified groups. *P. sativum* diversity shows partial overlap with *P. elatius* and is not geographically structured. A group of *P. elatius* accessions was identified as the suggested group from which *P. fulvum* and *P. abyssinicum* arose.

## Supplementary Materials

The following are available online at http://www.mdpi.com/2073-4425/9/11/535/s1, Table S1: List and description of analyzed material, Table S2: DARTseq dataset.
